# Weld Strength of Friction Welding of Dissimilar Polymer Rods Fabricated by Fused Deposition Modeling

**DOI:** 10.3390/polym14132582

**Published:** 2022-06-25

**Authors:** Chil-Chyuan Kuo, Jing-Yan Xu, Chong-Hao Lee

**Affiliations:** 1 Department of Mechanical Engineering, Ming Chi University of Technology, No. 84, Gungjuan Road, New Taipei City 243, Taiwan; u09217119@mail.mcut.edu.tw (J.-Y.X.); u09117014@mail.mcut.edu.tw (C.-H.L.); 2 Research Center for Intelligent Medical Devices, Ming Chi University of Technology, No. 84, Gungjuan Road, New Taipei City 243, Taiwan

**Keywords:** friction welding, weld strength, fused deposition modeling, flexural strength

## Abstract

Friction welding (FRW) is a promising method for joining cylindrical components of dissimilar and similar polymers or metals. In particular, FRW is capable of generating defect-free welds. Fused deposition modeling (FDM) has been widely employed in the automotive industry, ranging from lightweight tools, testing models, and functional parts. Conventionally, dissimilar parts fabricated by FDM are joined by glue. However, distinct disadvantages of this approach include both low joining strength and low joining efficiency. Hitherto, little has been reported on the characterizations of weld strength of FRW of dissimilar parts fabricated by FDM. In addition, FRW of dissimilar polymeric materials is a difficult task because different polymers have different physical, rheological, and mechanical properties. In this study, the effects of welding revolution on the weld strength of friction welding dissimilar parts fabricated by FDM are investigated experimentally. It was found that the average flexural strength of dissimilar polymer rods fabricated by FRW is about 1.52 times that of dissimilar polymer rods fabricated by gluing. The highest flexure strength can be obtained by FRW using polylactic acid (PLA) and PC (polycarbonate) rods. The average impact strength of dissimilar polymer rods fabricated by FRW is about 1.04 times that of dissimilar polymer rods joined by gluing. The highest impact strength can be obtained by FRW using PLA to PLA rods.

## 1. Introduction

Welding [1,2] is a process of joining materials, which is divided into solid-state and fusion welding. Friction welding (FRW) [3] is a non-fusion welding process that generates heat through mechanical friction between specimens in relative motion. FRW has potential applications in aerospace, automobile, automotive, chemical, railways, or marine industries because it is capable of producing good-quality leak-proof weld joints. In particular, FRW is a solid-state welding process that has some advantages, such as high efficiency, short welding time, and absence of shielding gas. Therefore, FRW is widely employed with thermoplastics or metals and in a wide variety application [4,5]. Kumaran et al. [6] investigated the effects of projection on the joint properties of FRW of tube-to-tube plate using an external tool. Results revealed that 1 mm projection has resulted in better strength compared to other weld conditions. The average weld interface Vickers hardness and weld strength are 70.58 and 84.72 MPa, respectively. Hynes et al. [7] developed a predicting thermal distribution model during the FRW of ceramics with metal using an aluminum interlayer for various time increments. It was found that the proposed simulation model provides the potential prediction of the formation of residual stress in the alumina-mild steel side of the interface, which leads to incomplete interlocking that results in poor joint strength. Azizieh et al. [8] studied the effects of FRW parameters on the microstructure and mechanical properties of K60 steel to ST37 steel joints. Tensile tests indicated that the strength of the weld zone is between those of the two components. The round head samples had better results than for the flat ones. Winiczenko et al. [9] investigated the effects of FRW parameters on the tensile strength and microstructural properties of dissimilar joints. It was found that tensile strength rises both with increasing friction time and friction force. The maximum tensile strength of friction-welded low carbon steel-ductile iron joints is 87% that of the base metal. Wang et al. [10] investigated the rotary FRW on dissimilar metals of aluminum and brass using an innovative pre-heating approach. The microstructure examination showed excessive intermetallic compound that has formed on the interface, indicating overheated temperature.

Additive manufacturing (AM) [11,12,13,14,15,16] is the computer-controlled sequential layering of materials to create three-dimensional physical models, which is particularly useful for the manufacture of prototypes or functional components with complex geometries. Fuse deposition modeling (FDM) is a common material extrusion (MEX) process. FDM [17,18] has been widely utilized for the production of prototypes, which is a process of fabricating physical models using materials such as polylactic acid (PLA) [19], polyamide (PA) [20], polycarbonate (PC) [21], or acrylonitrile butadiene styrene (ABS) [22,23,24]. Bernal et al. [25] utilized impulse excitation technique to obtain an effective isotropic Young’s modulus of FDM-printed thermoplastic materials used in topology optimization design. Results showed that greatest relative error of the measured frequencies with respect to the desired frequencies in the topology optimization problem is less than 2.9%. Lin et al. [26] demonstrated a versatile algorithm to produce isotropic products by optimizing the printing path. It was found that the workpiece was first separated into distinct areas in terms of the printing sequence, which increases the efficiency of the fabrication process. Paggi et al. [27] investigated specimens printed by the FDM method with corn starch and cellulose acetate. The results showed homogeneity and low porosity of the specimens printed at 230 °C and 90% flow rate. Camposeco-Negrete [28] optimized five responses associated with FDM process parameters. Results showed that the proposed method allowed for the simultaneous optimization of all the observed variables for the 3D printing process. Liu et al. [29] developed a novel rectangular-circular grid filling pattern of FDM in cellular lattice structures. Results showed that corresponding parameter settings and filling mode can improve mechanical performance and reduce material consumption.

Dissimilar welding of thermoplastic is one of the best solutions in engineering due to reduction in weight [30]. In general, FRW of dissimilar polymeric materials is a difficult task because the material flow during FRW is the factor most affecting the welding mechanism required for manufacturing qualified welded parts. In practice, physical models fabricated by FDM are joined by glue. However, two distinct disadvantages of this approach include low joining efficiency and low joining strength. In addition, the FRW of dissimilar polymeric rods is a difficult task because different polymers have different rheological and physical properties. Thus, proposing an efficient method to join physical models fabricated with high join strength is an important research topic. In this study, FRW is used to join dissimilar physical rods fabricated by FDM. Six different kinds of feedstock filaments are used to print weld specimens by FDM. An infrared thermal imager is used to monitor the peak temperature of weld joint during FRW. Three-point bending tests [31,32,33] and impact tests [34,35] are applied to evaluate the mechanical properties of the specimens after FRW. Optical microscopy, energy dispersive spectroscopy, and field-emission scanning electron microscopy were used to investigate the microstructure of welding zone. Finally, the mechanical properties of specimens after FRW are compared to those obtained by gluing.

## 2. Experimental Details

**Figure 1:**
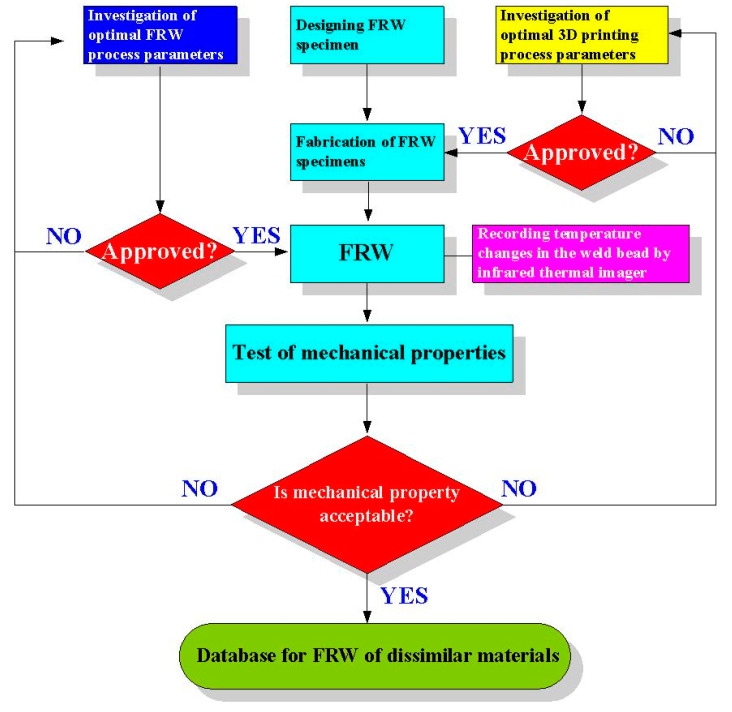
Flow diagram of the experimental methodology.

**Figure 2:**
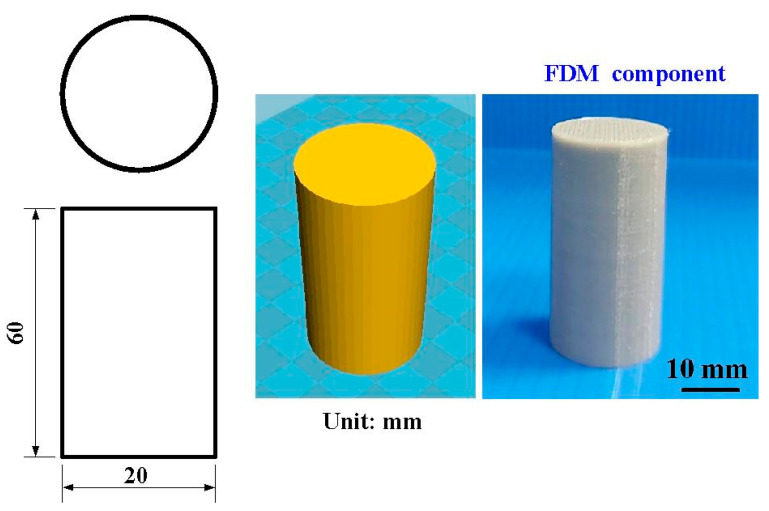
CAD model and dimensions of FRW specimen.

Figure 1 shows the flow diagram of the experimental methodology. The research process includes designing friction welding parts, investigating optimal 3D printing process parameters, and investigating optimal FRW process parameter. This flowchart has three judgment criteria. Judgment criterion one is whether the mechanical properties of FRW parts are acceptable. Judgment criterion two is whether the FRW parts can be welded. Judgment criterion three is whether the 3D part can be completely printed out. Figure 2 illustrates the CAD model and dimensions of the FRW specimen. The welding sample is a rod with a diameter of 20 mm and a length of 40 mm. Generally, the weld bead during FRW emits infrared energy [36]. In this study, Ultimaker Cura software was utilized to generate the 3D printing program. Six different kinds of feedstock filaments, i.e., PLA (Thunder 3D Inc., Hanoi, Vietnam), PLA filled with 10 wt.% glass fiber (GF) (Thunder 3D Inc.), PLA filled with 10 wt.% carbon fiber (CF) (Thunder 3D Inc.), ABS (Thunder 3D Inc.), PC, and PA were used to print FRW specimens using an FDM-based 3D printer (Infinity X1E, Photonier Inc., Ronkonkoma, NY, USA). The material costs of PLA, ABS, PLA + GF, PLA + CF, PA, and PC filaments were in New Taiwan dollar (NTD) at 1/g, 1/g, 1.5/g, 1.5/g, 2/g, and 1.5/g, respectively. The costs of a weld specimen built by the PLA, ABS, PLA + GF, PLA + CF, PA, and PC filaments were NTD 19, 20, 28.5, 30, 40, and 30, respectively. The chemical compositions of six different kinds of feedstock filaments were examined by an energy-dispersive X-ray spectroscopy (EDS) (D8 ADVANCE, Bruker Inc., Taipei, Taiwan). The process parameters for printing FRW specimens with PLA filament include a printing temperature of 200 °C, printing bed temperature at 60 °C, printing speed of 75 mm/s, and layer thickness of 0.1 mm. The infill density was fixed at 100%. The process parameters for printing FRW specimens with both PLA filled with 10 wt.% GF filaments include a printing temperature of 200 °C, printing bed temperature at 70 °C, printing speed of 75 mm/s, and layer thickness of 0.1 mm. The process parameters for printing FRW specimens with both PLA filled with 10 wt.% CF filaments include a printing temperature of 200 °C, printing bed temperature at 70 °C, printing speed of 75 mm/s, and layer thickness of 0.1 mm. The process parameters for printing FRW specimens with ABS filament include a printing temperature of 230 °C, printing speed of 45 mm/s, layer thickness of 0.1 mm, and printing bed temperature at 100 °C. The process parameters for printing FRW specimens with PA and PC filaments include a printing temperature of 245 °C, printing speed of 45 mm/s, layer thickness of 0.1 mm, and printing bed temperature at 100 °C.

**Figure 3:**
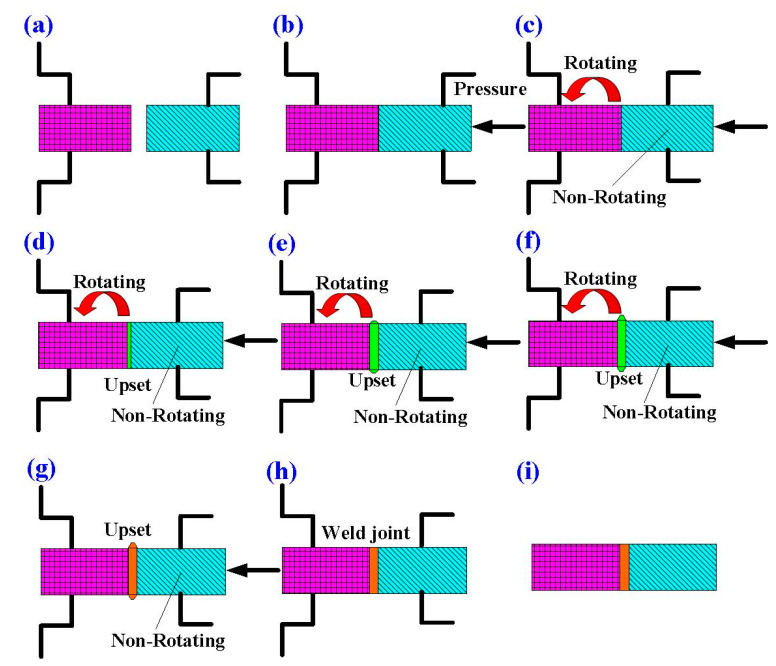
Schematic illustration of FRW process. (**a**) preparing two FRW specimens, (**b**) applying pressure to force FRW specimens into contact, (**c**) rotating one of the FRW specimens, (**d**) initial stage of FRW, (**e**) middle stage of FRW, (**f**) final stage of FRW, (**g**) FRW is completed, (**h**) the weld bead is processed, and (**i**) FRW is completed.

**Figure 4:**
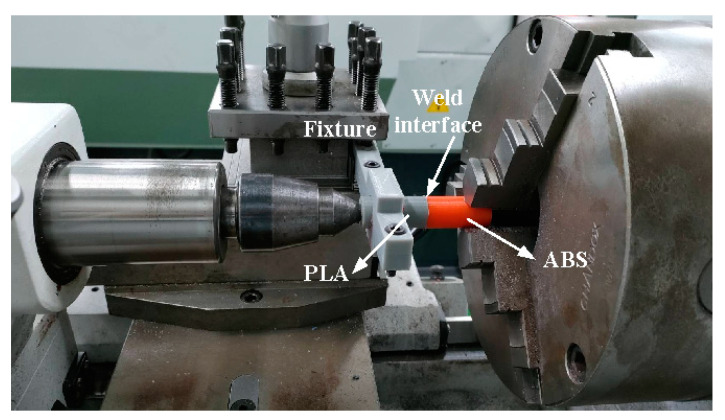
FRW of PLA to ABS rods.

FRW is a solid-state joining process that employs high axial pressure and rotational motion to generate frictional heat at the interface of joints. The friction pressure provides axial movement to obtain required weld strength. After FRW, upset pressure is for the consolidation of the weld. Figure 3 shows the schematic illustration of FRW process. One rod is held stationary while the other is rotated at a constant speed. Two rods are brought together under axial pressure for a certain period of time. The entire process of FRW has nine main steps, which involves (a) preparing two FRW specimens, (b) applying pressure to force FRW specimens into contact, (c) rotating one of the FRW specimens, (d) initial stage of FRW, (e) middle stage of FRW, (f) final stage of FRW, (g) FRW is completed, (h) the weld bead is processed, and (i) FRW is completed. In practice, rotational speed, friction time, friction pressure, upset pressure, and burn-off length are the most important parameters to be used in FRW process. To reduce human error, the fixed process parameters include rotational speed, total welding time, forge length, and times of forge. The cycle time of FRW is 60 s, which includes friction time, forge time, and cooling time is 30 s, 20 s, and 10 s. The burn-off length is 2.4 mm because the friction welding is performed 24 times with forge length of 0.1 mm each time. The rotation speed is kept constant at 650 rpm. The peak temperature of weld joint during FRW was recorded by an infrared camera (BI-TM-F01P, Panrico trading Inc., New Taipei City, Taiwan), which converts infrared energy into an electronic image that shows the apparent surface temperature of the weld bead during FRW. The impact strength test of the specimens was carried out with a Charpy impact testing machine with a pendulum length of 780 mm (780, Instron Inc., Norwood, MA, USA) [37]. The macrostructure and microstructure of welding zone was examined by an EDS, optical microscopy (OM) (Quick Vision 404, Mitutoyo Inc., Tokyo, Japan), and field-emission scanning electron microscopy (FE-SEM) (JEC3000-FC, JEOL Inc., Tokyo, Japan). To prevent one rod under pressure from rotating at the same time as a rotating rod, a fixture is designed to clamp one rod in this study. Figure 4 shows FRW of PLA to ABS rods. X-ray diffraction (XRD) analysis was performed to identify the phase obtained in the welding zone after FRW. The flexural strength of welded rods was investigated using a three-point bending test machine (RH-30, Shimadzu Inc., Kyoto, Japan) [38]. The movement speed of the bending test punch is about 1 mm/s. Flexural strength [39] can be determined by the following Equation (1). In this study, the length between two supports is 60 mm. The impact strength [40] can be estimated by using Equation (2):

(1) $$\sigma =\frac{8\mathrm{PL}}{{\pi d}^{3}}$$

where P is the axial load at the fracture point, d is the diameter of the welded rods, and L is the length between two supports:

E = WR [cos β − cos α)](2)

where W is the hammer mass, R is the distance from the impact point of specimen to rolling center, β is the finish angle after impact, and α is the start angle.

## 3. Results and Discussion

**Figure 5:**
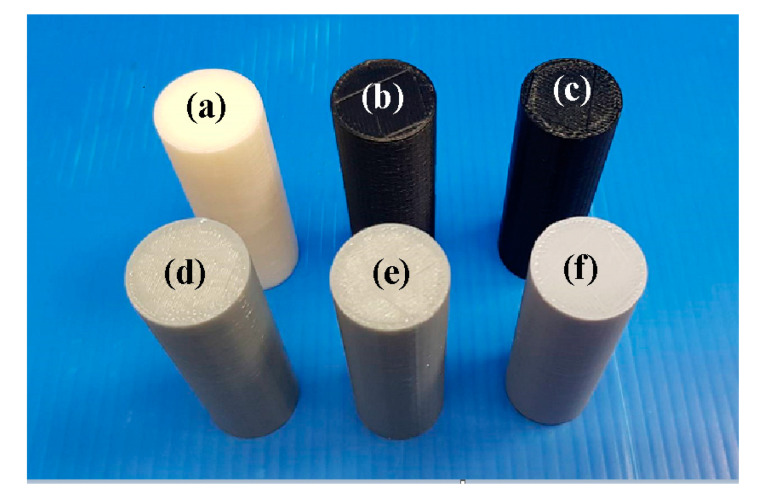
FRW specimens fabricated with (**a**) PLA, (**b**) ABS, (**c**) 10% GF reinforced PLA, (**d**) 10% CF reinforced PLA, (**e**) PA, and (**f**) PC feedstock filaments.

**Figure 6:**
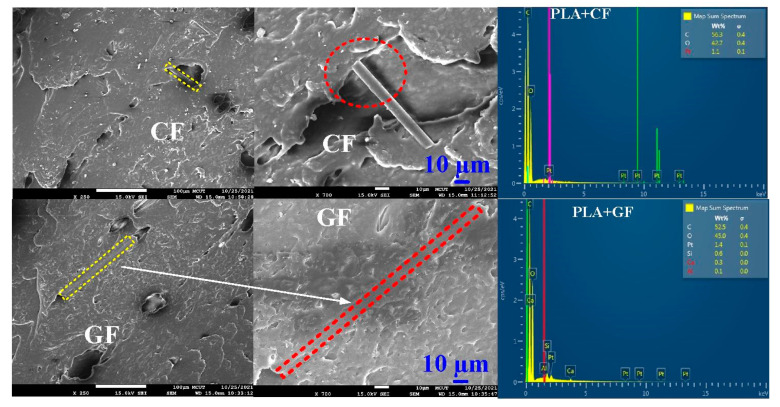
FE-SEM micrographs and chemical compositions of PLA filled with 10 wt.% GF reinforced PLA filled with 10 wt.% CF.

Figure 5 shows FRW specimens fabricated with PLA, ABS, 10% glass fiber (GF) reinforced PLA, 10% carbon fiber (CF) reinforced PLA, PA, and PC feedstock filaments. The EDS analyses were carried out for six feedstock filaments. Figure 6 shows FE-SEM micrographs and chemical compositions of a PLA filled with 10 wt.% GF reinforced PLA filled with 10 wt.% CF. It is interesting to note that the CF or GF was apparently found in the feedstock filaments applied to fabricate FRW specimens using AM technology. Notably, no impurity was observed. As can be seen, the major compositions of PLA filled with 10 wt.% CF are C and O. Note that the compositions of PLA filled with 10 wt.% GF are C, Si, O, Ca, and Al.

**Figure 7:**
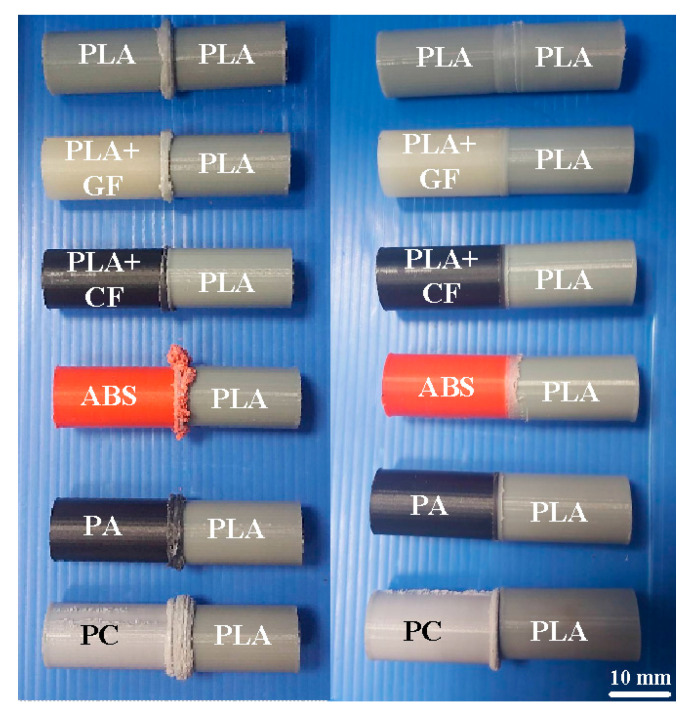
Results of FRW of dissimilar polymer rods.

**Figure 8:**
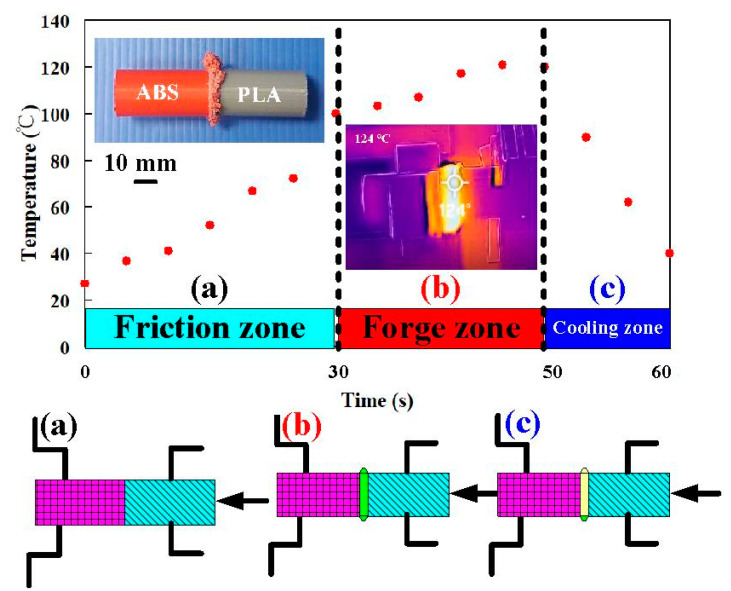
Changes of weld bead temperature as a function of time for three important stages of FRW (**a**) friction zone, (**b**) forge zone, and (**c**) cooling zone.

**Figure 9:**
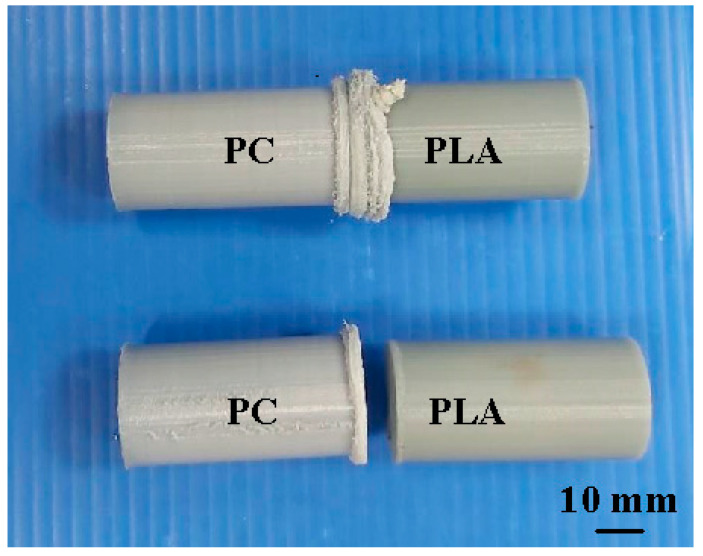
Failure of FRW of PLA to PC rods.

During FRW, the thermal energy at two faying surfaces is generated by the friction force. At the end of FRW, the compressive force is given to consolidate the weld and form a solid-state bond. Burn-off length is one of the significant process parameters for governing the heat generation and coefficient of friction during FRW. Burn-off length of 2.4 mm was used to study its effect on mechanical properties and weld interface characteristics in this study. The welding flash can then be removed by turning. Figure 7 shows the results of FRW of dissimilar polymer rods. During FRW, severe flashes were formed at the weld joints of the rods. As can be seen, PLA to PLA rods were welded successfully. Figure 8 shows changes of weld bead temperature as a function of time for three important stages of FRW. As expected, the temperature of weld bead increases from friction stage to forge stage and then decreases in the cooling stage. Figure 9 shows the failure of FRW of PLA to PC rods. The joint of PLA to PC rods can be separated easily due to both incorrect burn-off and incorrect rotation speed.

**Figure 10:**
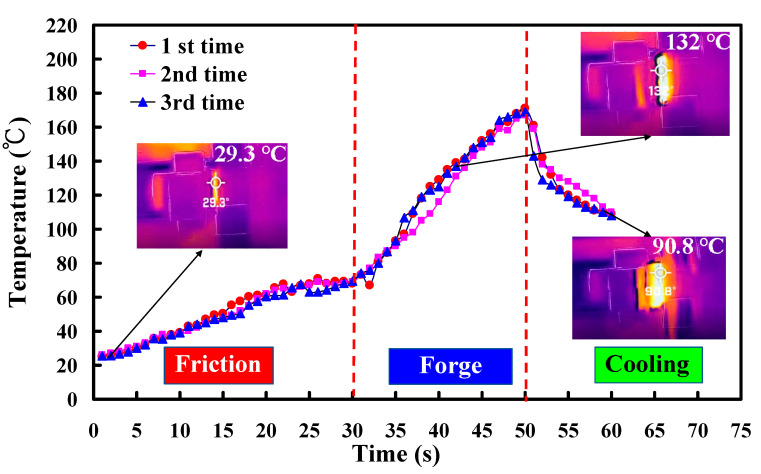
WI temperature as a function of welding time for the PLA to ABS rods in repeated experiments.

**Figure 11:**
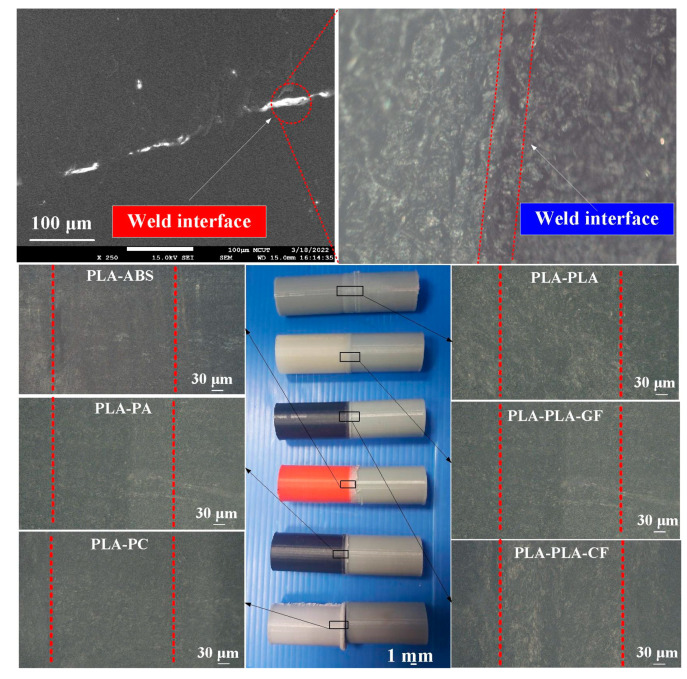
FE-SEM micrograph of the WI.

**Figure 12:**
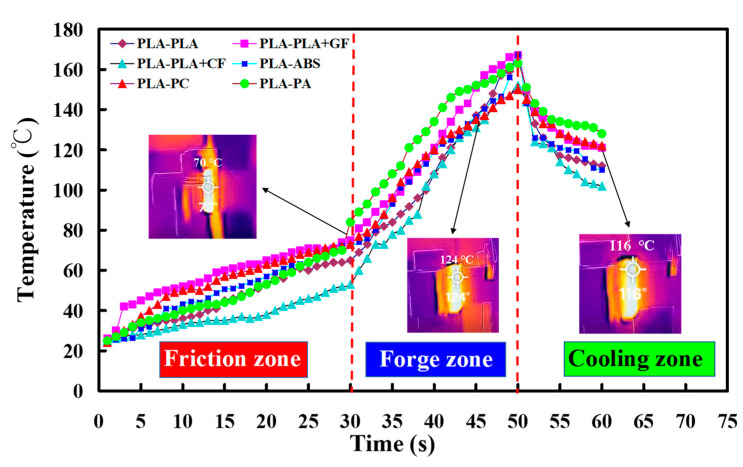
WI temperature as a function of welding time for six dissimilar joints.

The weld joint after FRW is characterized as a composite with three zones, i.e., interface, axial thermoplastic flow, and radial thermoplastic flow. The frictional heat (FH) during FRW can be estimated by the following Equation (3). μ, v, and p stand for the coefficient of friction, friction pressure, and rotational speed, respectively. Friction heat is positively related to the coefficient of friction, rotational speed, and friction pressure. To accurately measure the peak temperature of weld interface (WI) during FRW, an infrared thermal imager is used in this study. To reduce the influence of human factors on FRW, the experiment was repeated three times on PLA to ABS rods. Figure 10 shows the WI temperature as a function of welding time for PLA to ABS rods in repeated experiments. Two phenomena were found: (a) The relationship between WI temperature and welding time is repeatable and (b) the peak temperature of the bead is about 167–174 °C. Figure 11 shows the FE-SEM micrograph of WI. It should be noted that the bead width is very consistent, showing that the FRW result is acceptable. Figure 12 shows the WI temperature as a function of welding time for six dissimilar joints. As can be seen, the peak temperatures of WI for PLA to PLA rods, PLA to PLA filled with GF rods, PLA to PLA filled with CF rods, PLA to ABS rods, PLA to PC rods, and PLA to PA rods are approximately 167 °C, 167 °C, 152 °C, 167 °C, 150 °C, and 163 °C, respectively.

FH = μpv(3)

**Figure 13:**
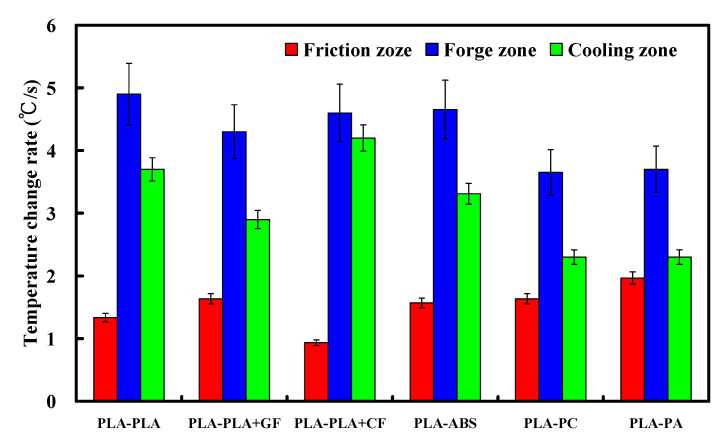
Temperature change rate of three zones in dissimilar joints.

**Figure 14:**
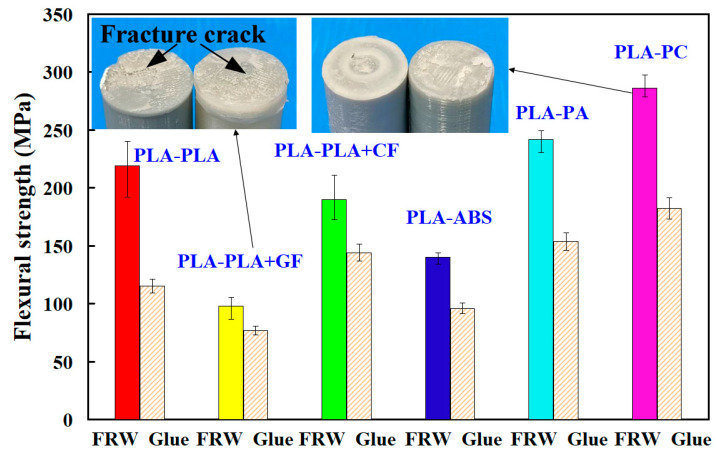
Flexure strength of dissimilar polymer rods fabricated by FRW and gluing.

Temperature change rate is the temperature change amount per unit time in each zone. Figure 13 shows the temperature change rate of three zones in dissimilar joints. Three phenomena were found: (a) friction zone—the highest temperature change rate is the FRW of PLA to PA rods with temperature change rate about 1.97 °C/s and the lowest change rate is the FRW of PLA to PLA/CF rods with a temperature change rate about 0.93 °C/s; (b) forge zone—the highest temperature change rate is the FRW of PLA to PLA rods with temperature change rate about 4.9 °C/s and the lowest temperature change rate is the FRW of PLA to PC rods with temperature change rate about 3.65 °C/s; (c) cooling zone—the highest temperature change rate is the FRW of PLA to PLA/CF rods with temperature change rate about 4.2 °C/s and the lowest temperature change rate is the FRW of PLA to PA rods with temperature change rate about 2.3 °C/s. In particular, frictional heating is the slowest, but cooling is the fastest for FRW of PLA to PLA/CF rods. Figure 14 shows the flexure strength of FRW of dissimilar polymer rods fabricated by AM. As can be seen, three phenomena were found. One is that the use of FRW to weld dissimilar polymer rods has a significantly better bending strength than the use of glue to join dissimilar polymer rods. The average flexural strength of dissimilar polymer rods fabricated by FRW is about 1.52 times of that of dissimilar polymer rods joined by gluing. The bending strengths for the FRW of PLA to PLA rods, PLA to PLA/GF rods, PLA to PLA/CF rods, PLA to ABS rods, PLA to PC rods, and PLA to PA rods are about 1.9, 1.28, 1.32, 1.46, 1.58, and 1.57 times that of dissimilar polymer rods joined by gluing, respectively. Another is that the highest weld strength can be obtained from FRW using PLA to PC rods. The other is that the weld strength of PLA to PLA/GF rods is not good, because chemical affinity is not easy to produce between two polymers [41]. As a result, the molecules of the two materials cannot be combined completely with each other. It is interesting to note that the weld strength of PLA to PC rods is the best because chemical affinity can be easily produced between two polymer rods. As a result, the molecules of the two materials can be combined with each other.

FRW led to a stronger bond because the process depends on inter-molecular bonding with the original material. Figure 14 shows the flexure strength of dissimilar polymer rods fabricated by FRW and gluing. Two typical fracture surfaces of dissimilar polymer rods after bending testing are shown in the inset. As can be seen, three phenomena were found. One is that the use of FRW to weld dissimilar polymer rods has a significantly better bending strength than the use of glue to join dissimilar polymer rods. The average flexural strength of dissimilar polymer rods fabricated by FRW is about 1.52 times of that of dissimilar polymer rods jointed by gluing. The bending strengths for the FRW of PLA to PLA rods, PLA to PLA/GF rods, PLA to PLA/CF rods, PLA to ABS rods, PLA to PC rods, and PLA to PA rods are about 1.9, 1.28, 1.32, 1.46, 1.58, and 1.57 times of that of dissimilar polymer rods jointed by gluing, respectively. Another is that the highest flexure strength can be obtained by FRW using PLA to PC rods. The other is that the flexure strength of PLA to PLA/GF rods is the lowest because chemical affinity is not easy to produce between two polymers [37]. As a result, molecules of the two materials cannot be combined completely with each other. It is interesting to note that the weld strength of PLA to PC rods is the best because chemical affinity is easy to produce between two polymer rods. As a result, the molecules of the two materials can be combined completely. 

The fracture failure morphology of the weld interface is shown in the inset. It should be noted that the morphology of the weld interface fracture surface is rough, with a large amount of micro-cracks [42]. Thus, the fracture crack [43] is subjected to the stress concentration caused by micro-defects.

**Figure 15:**
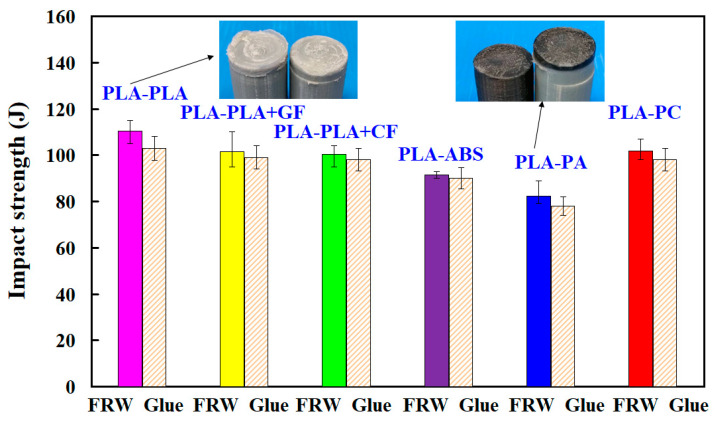
Impact strength of dissimilar polymer rods fabricated by FRW and gluing.

Figure 15 shows the impact strength of dissimilar polymer rods fabricated by FRW and gluing. Two typical fracture surfaces of dissimilar polymer rods after impact testing are shown in the inset. According to the results, two phenomena were found. One is that the use of FRW to weld dissimilar polymer rods has a slightly better impact strength than the use of glue to join dissimilar polymer rods. The average impact strength of dissimilar polymer rods fabricated by FRW is approximately 1.04 times of that of dissimilar polymer rods joined by gluing. The impact strengths for the FRW of PLA to PLA rods, PLA to PLA/GF rods, PLA to PLA/CF rods, PLA to ABS rods, PLA to PC rods, and PLA to PA rods are about 1.07, 1.02, 1.02, 1.02, 1.05, and 1.04 times that of dissimilar polymer rods joined by gluing, respectively. The other is that the highest impact strength can be obtained by FRW using PLA to PLA rods. However, the impact strength of PLA to PA rods is the lowest. This is attributed to the fact that the weld interface is brittle [44].

Based on the results described above, the findings of this study provide the greatest application potential in the industry because this method is very practical and can be employed to join fluid mechanical components [45], transmission shafts [46], aircraft components, aerospace components, automotive components, or axle shafts using FRW. To further evaluate the tensile strength of FRW of dissimilar polymer rods fabricated by AM technology, test specimens can be prepared according to standards from ASTM International [47,48,49]. Thus, the research results can provide more application value in the industry. In addition, molecular orientation in the joints after FRW can also be investigated by differential scanning calorimetry [50]. In this study, the weld specimens were prepared by FDM. A polymer rod with dissimilar materials can also be prepared by the MEX method directly with a dual nozzle device. The difference in the mechanical properties between the two methods is also an interesting research topic. In this study, FRW was carried out in the atmosphere. The FRW of dissimilar materials with shield gas is also an interesting research topic. These issues are currently being investigated, and the results will be presented in a later work.

## 4. Conclusions

FDM is a promising 3D printing technology by means of which functional physical models with a variety of thermoplastics can be fabricated economically and swiftly. FRW is a solid-state joining process that is effectively employed for joining similar or dissimilar materials. Dissimilar joining of thermoplastic is very attractive in the various industries because of the reduction in weight. It should be noted that the FRW of dissimilar polymeric materials is a difficult task since different polymers have different thermal and mechanical properties. In this study, the characterization of weld strength of FRW of dissimilar cylinders fabricated by FDM is investigated experimentally. The temperature of the joint interface during the welding process was monitored. OM and FE-SEM were employed to investigate the microstructure of weld joints. The main conclusions from the experimental work in this study are as follows:

1. The remarkable findings in this study are very practical and provide potential applications in the research and development stage because this technique can be used to fabricate functional components for functional testing in the industry.
2. FRW to weld dissimilar polymer rods has a significantly better bending strength than the use of glue to join dissimilar polymer rods.
3. The average flexural strength of dissimilar polymer rods fabricated by FRW is about 1.52 times of that of using glue to join dissimilar polymer rods. The highest flexure strength can be obtained by FRW using PLA to PC rods.
4. The average impact strength of dissimilar polymer rods fabricated by FRW is about 1.04 times of that of dissimilar polymer rods jointed by gluing. The highest impact strength can be obtained by FRW using PLA to PLA rods.

## Data Availability

Data and materials are available.
